# Time‐Resolved Product Observation for CO_2_ Electroreduction Using Synchronised Electrochemistry‐Mass Spectrometry with Soft Ionisation (sEC‐MS‐SI)

**DOI:** 10.1002/anie.202312607

**Published:** 2023-10-24

**Authors:** Guohui Zhang, Anthony Kucernak

**Affiliations:** ^1^ Department of Chemistry Imperial College London White City Campus London W12 0BZ United Kingdom; ^2^ State Key Laboratory of Catalysis Dalian Institute of Chemical Physics Chinese Academy of Sciences Dalian 116023 China

**Keywords:** Electrochemical CO_2_ Reduction, Kinetics, Operando Mass Spectrometry, Reaction Mechanisms, Soft Ionisation

## Abstract

The mechanistic understanding of electrochemical CO_2_ reduction reaction (CO_2_RR) requires a rapid and accurate characterisation of product distribution to unravel the activity and selectivity, which is yet hampered by the lack of advanced correlative approaches. Here, we present the time‐resolved identification of CO_2_RR products by using the synchronised electrochemistry‐mass spectrometry (sEC‐MS). Transients in product formation can be readily captured in relation to electrochemical conditions. Moreover, a soft ionisation (SI) strategy is developed in MS for the direct observation of CO, immune to the interference of CO_2_ fragments. With the sEC‐MS‐SI, the kinetic information, such as Tafel slopes and onset potentials, for a myriad of CO_2_RR products are revealed and we show the hysteresis seen for the evolution of some species may originate from the potential‐driven changes in surface coverage of intermediates. This work provides a real‐time picture of the dynamic formation of CO_2_RR products.

## Graphical abstract

The dynamic evolution of electrochemical CO_2_ reduction (CO_2_RR) products is unambiguously elucidated with high temporal resolution using the synchronised electrochemistry‐mass spectrometry with soft ionisation (sEC‐MS‐SI), where CO species can be detected without the interference of CO_2_ fragmentation, providing insights about the reaction mechanism and kinetics for CO_2_RR.

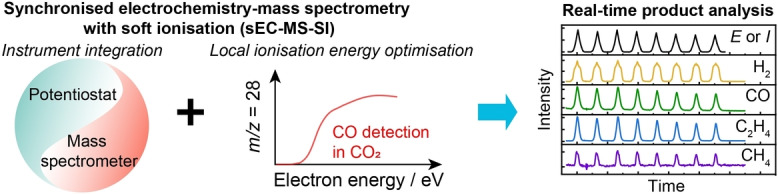

## Introduction

Electrochemical CO_2_ reduction reaction (CO_2_RR) powered by renewable sources has been regarded as a promising route to mitigate the environmental and energy issues across the world, which is pivotal to establish a carbon neutral society.[^1^, ^2^, ^3^] The reaction can be manipulated to generate a wide range of products via multiple proton‐coupled electron transfer steps, subject to several factors, such as catalysts, potentials/currents and electrolytes.[^4^, ^5^] To explicitly describe the dynamic evolution in CO_2_RR, real‐time product analysis offers significant advantages, which can further provide guidelines for rationalising the reaction design towards targeted products. However, this step poses enormous technical challenges to the conventional methods, such as nuclear magnetic resonance (NMR) spectroscopy, high‐performance liquid chromatography (HPLC) and gas chromatography (GC), which are usually operated in an ex situ manner requiring periodic sampling followed by a discrete analysis time sequence.[^6^, ^7^, ^8^, ^9^] Owing to the time involved in performing the measurements, and the sampling protocols, the results from these sorts of studies do not provide real‐time product distribution, not to mention that the CO_2_RR is highly susceptible to electrocatalyst degradation.[^10^, ^11^] Hence, it is imperative to explore high resolution characterisation platforms for simultaneously visualising CO_2_RR product species.

Operando approaches that couple various analytical techniques to correlate the information collected from different perspectives under working conditions are capable of capturing the intrinsic picture of electrochemical reactions, and suitable for tracking the product evolution and distribution in real time.[^12^, ^13^] In the past decades, the device designs represented by the development of gas diffusion electrodes (GDEs) have greatly advanced CO_2_RR research.[^14^, ^15^] Collection of gas‐phase species from these systems can usually be combined with mass spectrometry (MS) to elucidate the gaseous and volatile species, characteristic of high sensitivity and fast response (on the second's timescale, superior to the min's resolution of GC). To this end, operando/in situ electrochemical MS, such as differential/online electrochemical mass spectrometry (DEMS/OLEMS), have been developed.[^16^, ^17^, ^18^, ^19^, ^20^, ^21^] These hyphenated measurements have been shown to deliver a comprehensive understanding of the local reaction environment, enabling the structure/composition‐activity relationship to be assessed. Nonetheless, it should be noted that the study of CO_2_RR by MS is still hindered by several issues. Firstly, formation of bubbles at the electrochemical interface, and transport of gases from the electrode to the MS cause possible delays in product analysis, which need to be carefully estimated. Secondly, it is difficult to accurately correlate the datasets from separate instruments on the same time axis, due to the existing time delay (between product generation and detection) and the discrepancy in data collection rates. Although some systems with third‐party data acquisition platforms (i.e., LabView) have been reported,[^22^, ^23^] they require enormous effort in intricate hardware communications and software programming, as well as expensive accessories. Thirdly, the detection of CO product is severely complicated by the fragmentation of CO_2_ molecules during the ionisation process of MS, impeding the reliable and complete quantification of reaction products.

Here, we investigate the dynamic evolution of CO_2_RR products in a three‐fold strategy by using (i) the gas accessible membrane electrode (GAME), which allows for high rate gas‐evolving reactions free from bubble formation; (ii) the synchronised electrochemistry‐mass spectrometry (sEC‐MS): this instrumental coupling, based on physical communications between the potentiostat and the mass spectrometer, allows for the concurrent display and alignment of two set of signals in the MS software; and by developing (iii) the soft ionisation (SI) protocol, where the fragmentation of CO_2_ in MS is selectively controlled, enabling CO evolution to be directly evaluated. As a result, Tafel slopes are extracted from the synchronous faradaic current and MS response to show the kinetic feature of CO_2_RR. The onset potentials for the CO_2_RR products on Cu are found to be in an order of H_2_>CO>C_2_H_4_>CH_4_. Further, the hysteresis seen during the product formation can be accounted for by the potential‐dependent variation in surface coverage of key intermediates (e.g., CO*).

## Results and Discussion

**Figure 1:**
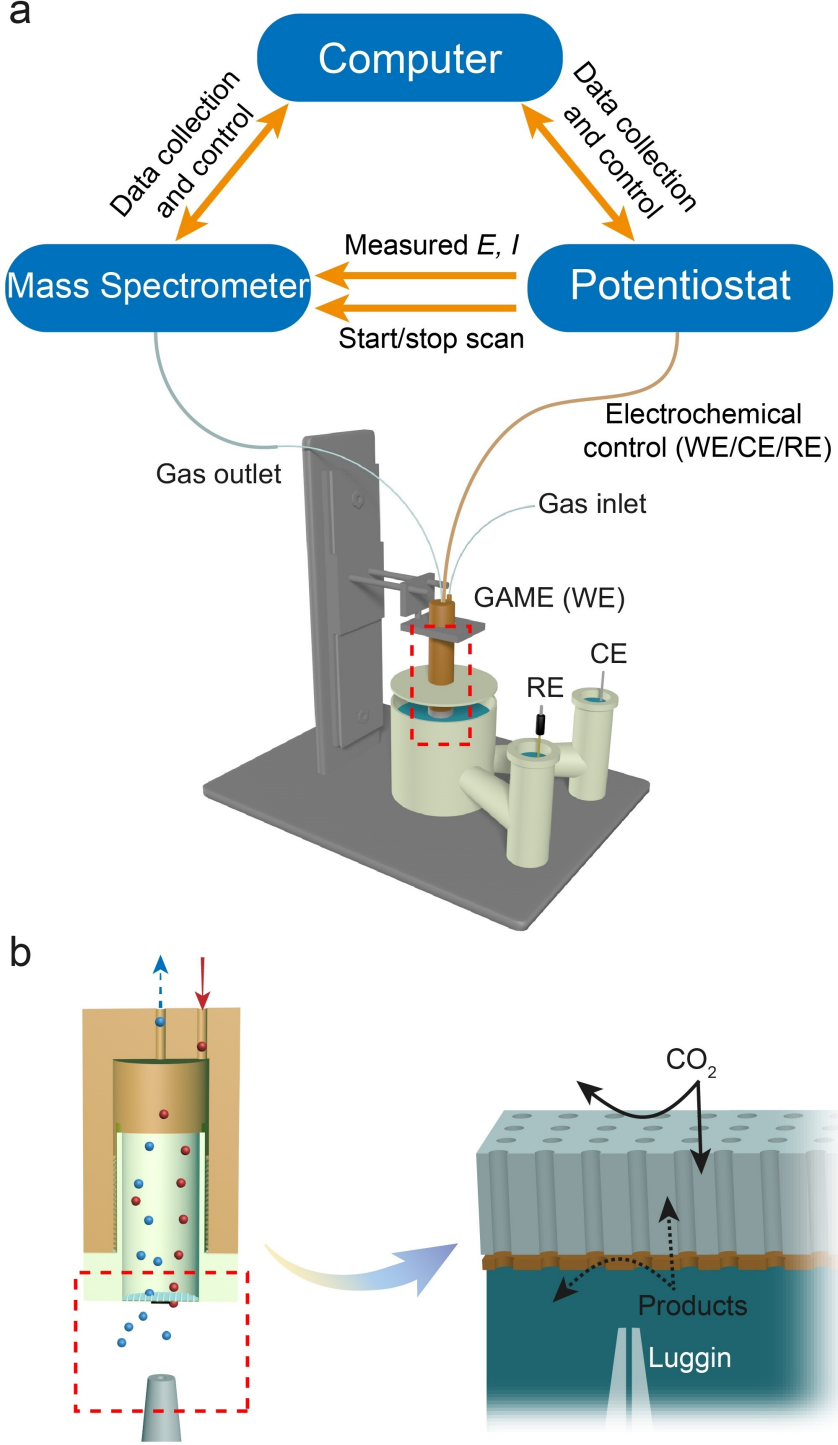
(a) Schematic for the configuration of the sEC‐MS‐SI platform. The lower section shows the GAME setup for CO_2_RR measurements. (b) Slice view of the GAME configuration (outlined area in a) composed of an enclosed gas chamber and the membrane electrode, and a further illustration shows the three‐phase reaction interface, associated with a gas delivery (solid arrows) and collection (dashed arrows) protocol.

Synchronous electrochemical‐mass spectrometric measurements are performed by feeding analogue output voltages representing the current and potential measured by the potentiostat to spare analogue inputs of the MS system. Then, the MS system records both electrochemical current/voltage and partial pressures associated with the ionisation process of incoming species (Figure 1a). The MS system is set to start acquisition upon receiving a trigger signal from the potentiostat, indicating that an electrochemical measurement is being performed. Further details are provided in the Supporting Information (Figure S1 and Table S1 in Section S1).

In addition to the instrumental connections, the in‐house designed GAME system was implemented to study the CO_2_RR (Figure 1b). This configuration has been shown to significantly promote mass transport and facilitate the development of a range of correlative approaches in our previous work.[^24^, ^25^] The approach is useful in studying gas evolving reactions, as the electrodes allowing for high‐rate transport of gaseous products enable the measurements to be operated at high current density without the formation of bubbles. Bubbles formation is deleterious as they can act as a reservoir of products, leading to complexities in correlating product formation rate with faradaic current. The Au‐sputtered polycarbonate track etch (Au/PCTE) membrane was first chosen as a model catalyst for the CO_2_RR study herein, due to the relatively simple product distribution, mainly comprising H_2_ and CO in the gas phase.[^26^, ^27^] The GAME is brought into contact with the electrolyte in a gas‐tight cell via a custom‐made holder, and the efficient gas‐circulating pathways equipped within the GAME can enable a rapid gas delivery of CO_2_ to the electrode surface (functionalised as a GDE). Subsequently, the CO_2_ gas can diffuse through the linear channels (tortuosity=1) of the thin (≈12 μm) porous membrane, establishing an efficient three‐phase (gas‐electrode‐electrolyte) boundary, where the electrocatalytic CO_2_RR can readily occur. Note that this electrode structure is more than one order of magnitude thinner than the electrode supports used in other GDEs.^[24]^ At the same time, the gaseous and volatile products generated at the interface will migrate back to the GAME, with a very short diffusion time due to the limited amount of solution‐based diffusion required (<50 nm length). Once in the GAME chamber, the products are collected and then transported by the excess CO_2_ flow to the tandem MS in a rapid fashion. Therefore, this design ensures a fast dual‐way mass transport pattern for both the reactant and products through the membrane electrode over the course of the reaction, and opens up new routes for real‐time product analysis.

However, regarding characterisation of the CO_2_RR by the MS technique, a common obstacle is that the fragmentation of CO_2_ under normal circumstances (i.e., with a defaulted electron energy of 70 eV to ensure a complete ionisation; Figure S2 in the Supporting Information) would lead to a notable background at the mass‐to‐charge ratio (*m/z*) of 28 (CO^+^), where the CO product is monitored. This signal overlap makes it difficult to deconvolute the electrochemically produced CO from the fragments of CO_2_, let alone the quantification of CO species. To mitigate this effect, the contribution of CO_2_ fragmentation to the *m/z*=28 signal is conventionally accounted for by subtracting a value deduced from the ratio of *m/z*=28 and *m/z*=44 under CO_2_ atmosphere.[^16^, ^28^] However, large errors and uncertainties, and limited ability to measure low levels of CO are associated with this method, as the noise in the CO_2_ signal is considered in the inferred CO signal, and the CO^+^ from the electrochemically produced CO will only be a small fraction of the total amount due to the large excess of CO_2_.^[8]^ Furthermore, the CO_2_ cracking pattern has to be carefully measured for each specific configuration, as the ratio of *m/z*=28 and *m/z*=44 is highly dependent on the mass spectrometers and parameter settings, and may even vary with time as the instrument operates. To this end, it is extremely meaningful to exploit an approach to inherently eliminate the CO_2_ fragmentation at *m/z*=28 to ensure the direct measurement of CO from CO_2_RR.

**Figure 2:**
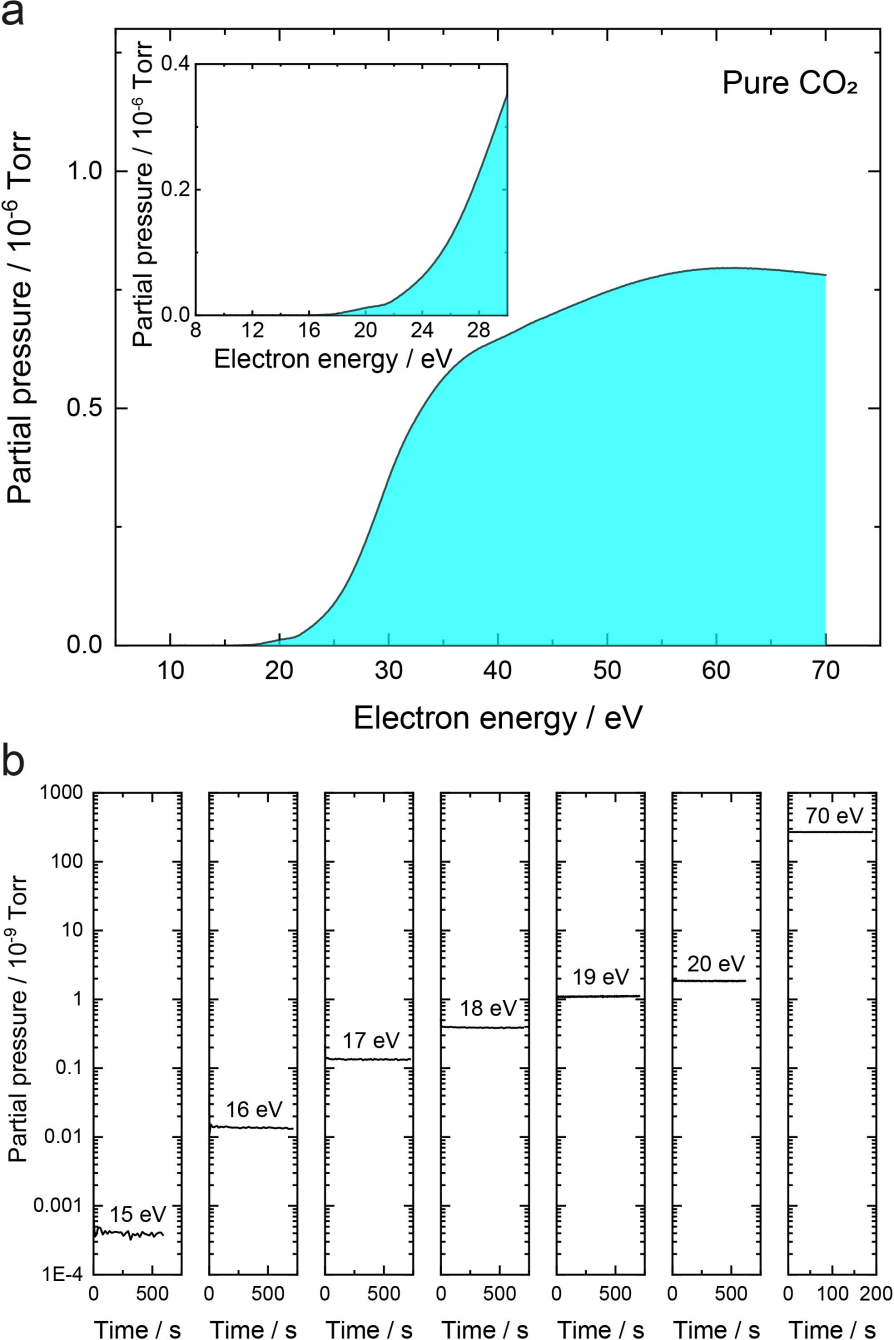
(a) Plot of MS signal at *m/z*=28 (CO^+^) as a function of electron energy continuously varied in the MS under a pure CO_2_ flow. The profile of the low energy region is shown in the inset. (b) Dependence of the MS response at *m/z*=28 (CO^+^) on the electron energy of the MS under CO_2_ atmosphere.

In this work, the mass spectrometric response at *m/z*=28 was first monitored under a flow of CO_2_, while the electron energy of MS used to ionise the gaseous species was constantly varied. As demonstrated in Figure 2a, only a negligible level of signal at *m/z*=28 is observed until the electron energy exceeds 18 eV, and then the signal increases drastically. Beyond the energy of ca. 35 eV, the intensity asymptotically reachs a steady value. This illustrates that CO_2_ fragmentation is enhanced with the increased electron energy of MS. The fragmentation behaviour of CO_2_ is further investigated in individual measurements with a range of energy values, and the results are shown in Figure 2b. When the energy is changed from 15 eV to 70 eV, the *m/z*=28 response is increased by almost 6 orders of magnitude. However, there is only a slight change from 15 eV to 20 eV. These results highlight the possibility of manipulating the effect of CO_2_ fragmentation on the detection of CO by locally adjusting the energy for ionisation, allowing the recorded *m/z*=28 response to be solely assigned to the CO product, in accordance with a recent work.^[8]^ Of note, the electron energy cannot be kept too small, which would otherwise cause the insufficient ionisation (Figure S3), considering the ionisation energy of CO is ca. 14 eV,^[29]^ thereby lowering the signal towards the detection limit of the instruments. It should be also noted that the *m/z*=28 signal starts to increase immediately after 17.3 eV under N_2_ (acting as a comparative atmosphere to CO_2_ for electrocatalysis herein; see Figure S4 of the Supporting Information). Therefore, an electron energy of 17 eV seems to be suitable to minimise the fragmentation of CO_2_ and N_2_, while the ionisation of CO is guaranteed. In the following measurements, an analysing environment of an electron energy of 17 eV is set at *m/z*=28 for the deconvolution of CO, while the rest of the *m/z* values are still monitored using 70 eV, in a local SI regime. Note that this is intrinsically different from the previous protocol,^[8]^ where a lower electron energy was applied for the observation of all the species, leading to a universal reduction in ionisation probablity and signal intensity.

**Figure 3:**
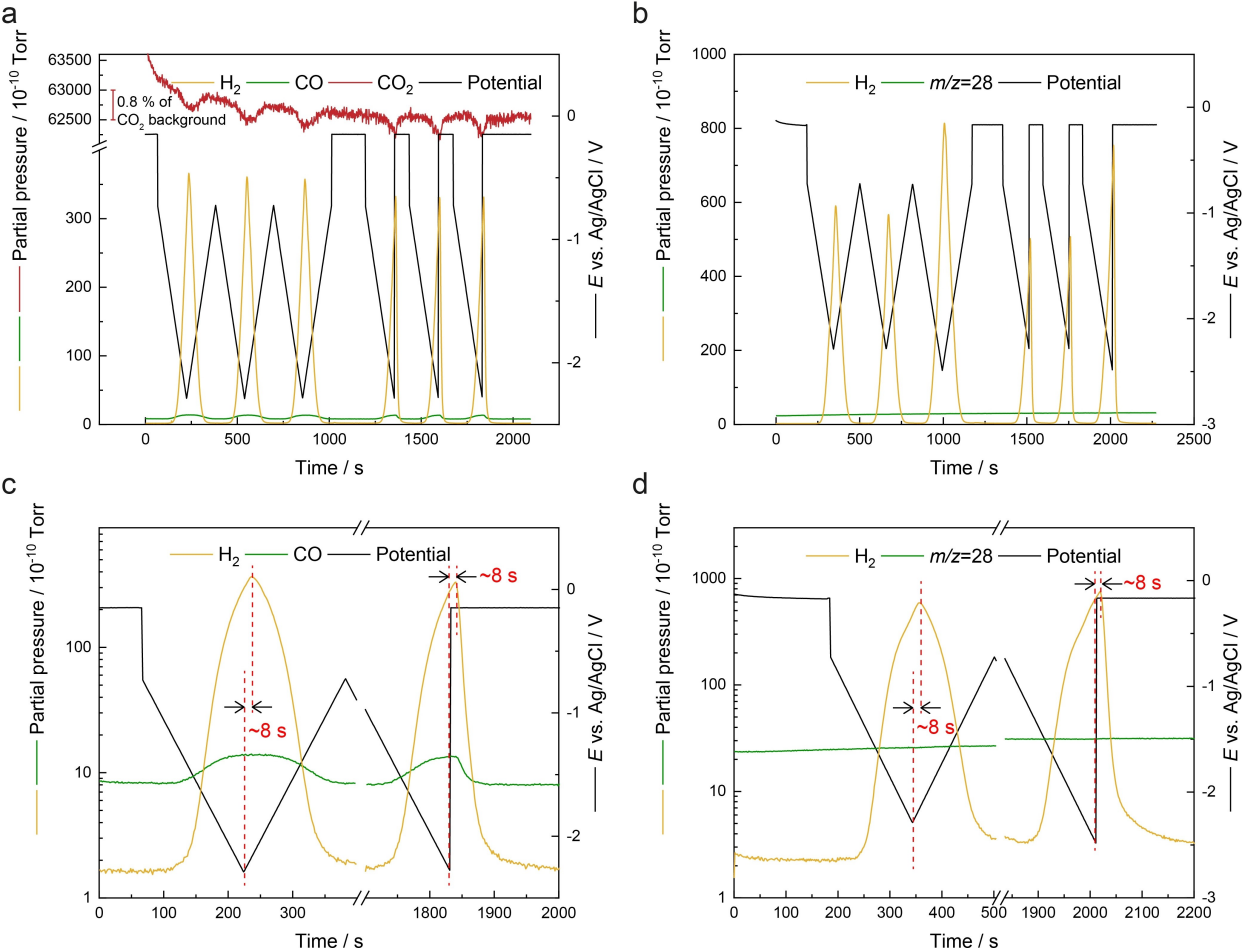
Mass spectrometric responses of *m/z*=2 (H_2_), 28 (CO or N_2_) and 44 (CO_2_) and the synchronised potential profiles (without iR correction) during 3 CVs and 3 LSVs of Au/PCTE under (a) CO_2_ and (b) N_2_ atmosphere, respectively. Scan rate: 10 mV s^−1^. Note in (b), the potential ranges were extended in the 3^rd^ CV and 3^rd^ LSV to match the current scale obtained in CO_2_. Enlarged regions of (a) and (b) are shown in (c) and (d), respectively, illustrating the profiles of the 1^st^ CV and the 3^rd^ LSV. The peak positions are indicated by red dashed lines.

Next, a series of cyclic voltammograms (CVs) and linear scan voltammograms (LSVs) of Au/PCTE were carried out in 0.5 M KHCO_3_ with the sEC‐MS‐SI in the presence and absence of CO_2_, respectively. As seen in Figure 3 and Figure S5, the electrochemical and mass spectrometric results are well resolved in the same plots, as a result of the instrumental and signal synchronisation scenario discussed above and in the Supporting Information. Under CO_2_ atmosphere (Figure 3a), periodic changes are observed in the MS signal of CO_2_ (*m/z*=44) during the electrochemical scans, indicative of the consumption of CO_2_ by the electrocatalytic reaction at the GAME, and the dilution of the CO_2_ by gaseous products (H_2_ and CO). Meanwhile, the MS responses for H_2_ (*m/z*=2) and CO (*m/z*=28) are deconvoluted, which reach peak values at the same time, slightly behind the points where the voltammetry changes scan direction (for CVs) or terminates (for LSVs). Both the maxima values of H_2_ and CO signals are comparable to their counterparts between the two voltammetry techniques. The slight difference seen in the enlarged region shown in Figure 3c can be ascribed to the minor discrepancy in faradaic currents (Figure S6). The data synchronisation further enables the delay time for the sEC‐MS‐SI configuration to be directly assessed from the LSV results, since any lag in product analysis would be visualised after the removal of potential at the end of electrochemical measurements. The delay time is found to be generally ca. 8 s, albeit with small variations in different scans due to the sampling rate (≈1.7 s per data point) adopted in the measurements. Interestingly, the time difference between the peaks of potentials and MS responses from the CVs is also ca. 8 s, implying the negative potential limits of CVs should be temporally colocated with the MS peaks.

When the feed gas is switched to N_2_ in the GAME (Figures 3b and 3d), only H_2_ is seen in the MS spectra, even when the potential region is intentionally extended to more negative limits (in the 3^rd^ CV and 3^rd^ LSV) to produce the same scale of current as the case in CO_2_. A background signal at *m/z*=28 is stable during the voltammetry, due to the ionisation of miniscule amount of N_2_. Again, the delay time is deduced to be ca. 8 s from the comparison of CVs and LSVs with MS results (Figure 3d; the corresponding voltammograms are shown in Figure S7), in line with the measurements performed in CO_2_.

**Figure 4:**
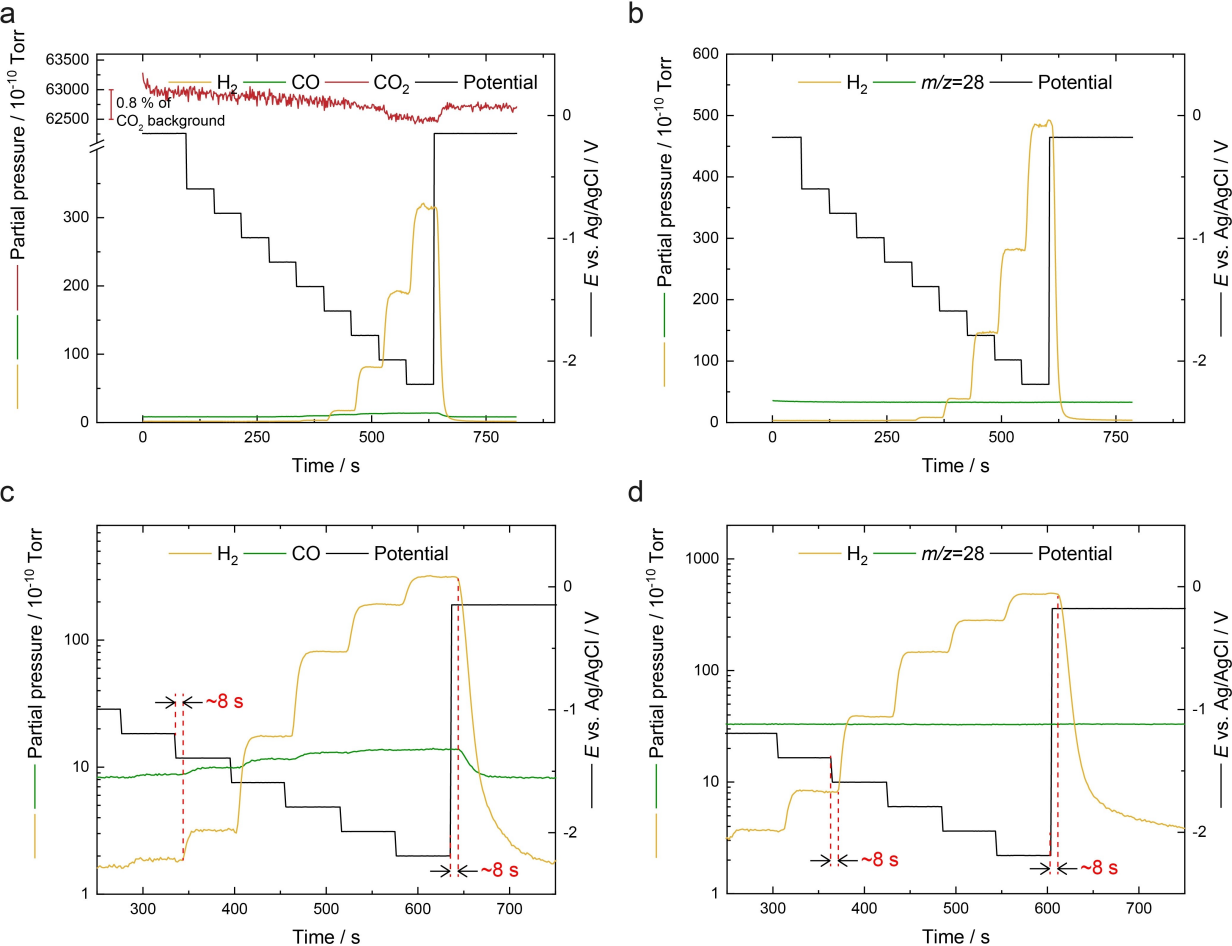
Mass spectrometric responses of *m/z*=2 (H_2_), 28 (CO or N_2_) and 44 (CO_2_) and the synchronised potential profiles (without iR correction) during chronoamperometry of Au/PCTE under (a, c) CO_2_ and (b, d) N_2_ atmosphere, respectively. The transition points of the steps are indicated by the dashed red lines.

Chronoamperometry measurements were also performed with the sEC‐MS‐SI to further interrogate the CO_2_RR behaviour of Au/PCTE at steady state. In the presence of CO_2_ (Figures 4a and 4c), the concentration of CO_2_ maintains at a relatively constant level in the beginning. As the potential decreases stepwise, the MS response of CO_2_ starts to drop due to the consumption and dilution effects previously mentioned, and meanwhile the H_2_ and CO species appear on the mass spectra. During the individual potential steps, the current is almost stable (Supporting Information, Figure S8), as well as the MS responses of H_2_ and CO. Note that the derivative of H_2_ response reaches a maximum at 8 s after the application of potential and decays to 0 after 22 s, at which point steady state has been established (Figure S9), in line with the DEMS results.^[30]^ The stability of MS response reiterates the fact that mass transport is fast in this system and not affected by solution diffusion processes or possible bubble formation. When the supply gas is changed to N_2_, only H_2_ production is tracked (Figures 4b and 4d). Under N_2_ (Figure 4d), there is an unchanging background signal of ≈3×10^−9^ Torr at *m/z*=28, due to the very small amount of ionisation of N_2_ in the SI process. This background level is about four times greater than that under CO_2_ (≈8×10^−10^ Torr, Figure 4c). Hence, care must be taken when using N_2_ as a sweep gas even if soft ionisation is adopted, as it will raise the background level and may make small changes in CO production difficult to measure. In our case, we use CO_2_ as the sweep gas for CO production, which has a lower background of *m/z*=28 signal. The delay time is consistently estimated to be ≈8 s from the enlarged areas of the MS signals shown in Figures 4c–d, which agrees well with the voltametric data above. These reproducible results indicate that the GAME‐based configuration is free from the accumulation of gas bubbles (despite using static electrolyte), which again highlights the efficient gas transport in our system.

**Figure 5:**
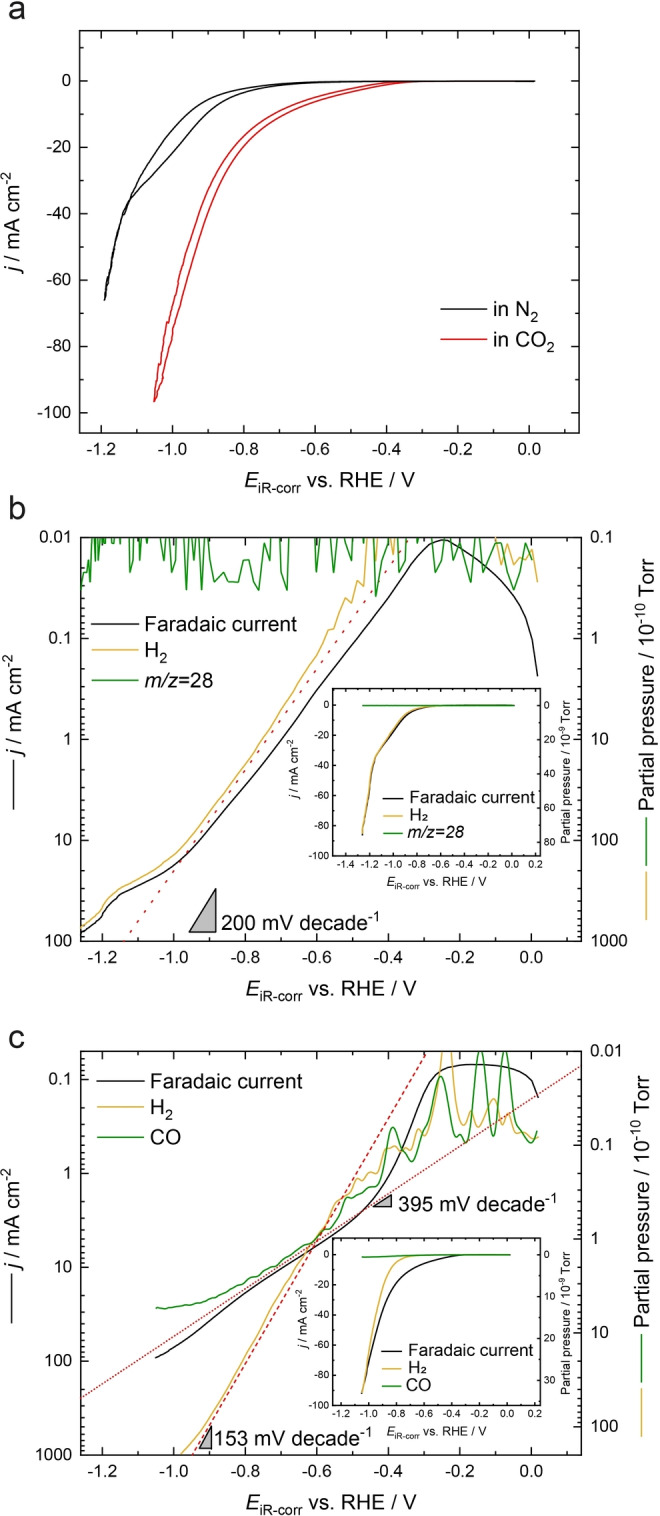
(a) CVs of Au/PCTE recorded in N_2_ (black) and CO_2_ (red) at a scan rate of 10 mV s^−1^. (b–c) LSVs (recorded at 10 mV s^−1^) and the corresponding MS responses of H_2_ (*m/z*=2) and CO (*m/z*=28) in N_2_ and CO_2_, respectively on logarithmic y‐axis scales. The plots on linear‐y‐scales are shown in the insets. Indicative lines with Tafel slopes are plotted over the data.

Next, the transient kinetic information about the CO_2_RR is explored from the sEC‐MS‐SI results. As seen from Figure 5a, the CVs of Au/PCTE show distinct behaviours under N_2_ and CO_2_.

Under CO_2_, a more positive onset potential is seen, and higher current densities are obtained at the same potentials, in comparison to those under N_2_. Relatively little hysteresis is observed between the opposite scan directions, suggesting that the system equilibrates faster than the scan rate to maintain pseudo‐steady state at all potentials. As demonstrated by the aforementioned parts, this scenario of sEC‐MS‐SI can eliminate the time delay by aligning the data from both instruments, especially using the LSV technique. Therefore, the sEC‐MS‐SI technique enables both the electrochemical and MS signals to be concurrently plotted against the electrode potentials. The LSVs and associated MS responses are shown in Figures 5b–c, while the CVs and the corresponding MS responses are displayed in Figures S10 and S11 of the Supporting Information. In Figures 5b–c, the logarithmic y‐axes covering 4–5 orders of magnitude in electrochemical current/MS response are used to better understand the transitions between different reaction domains. The ranges of the faradaic current and MS y‐axes are chosen to be equal (a factor of 10,000 in Figure 5b and 20000 in Figure 5c) to allow direct comparison of the respective variations. Inset in each of the figures is the corresponding plot with a linear y‐axis (enlarged view of the inset of Figure 5c is shown in Figure S12). For both of these results, the average backgrounds at *m/z*=2 and *m/z*=28 (and faradaic current) have been subtracted from the respective signals in order to provide information about the net production of the relevant species. In N_2_ (Figure 5b), both the hydrogen (*m/z*=2) and faradaic current signal show an onset potential close to −0.25 V (vs. RHE), obtained through back‐interpolating the respective take‐off curves. It is found that the MS signal of H_2_ follows the faradaic current very well across the entire current range, showing a clear Tafel slope of 200 mV decade^−1^ over two orders of magnitude in both current and MS response. The close coupling of MS and faradaic current shows that only the hydrogen evolution reaction (HER) is occurring on the electrode and no signal is seen for *m/z*=28, as might be expected. The ability to derive such a clear Tafel slope over two orders of magnitude in faradaic current/MS response attests to the fast mass transport of our system. Beyond the current density of 10 mA cm^−2^, there is an increase in Tafel slope suggesting a possible mechanistic change or other effect.

When the atmosphere in the GAME is switched to CO_2_ (Figure 5c), the response is significantly changed. The faradaic current shows two inflection points—one at about −0.25 V (vs. RHE) showing a rapid increase in the magnitude of current above the background, and a second at −0.45 V (vs. RHE) where the current deviates and the effective Tafel slope increases. The faradaic current also seems to be larger in the potential range −0.4 to −0.8 V than either of the mass spectra traces (inset plot), although it follows both of them in the logarithmic plot. We attribute this difference to faradaic processes not producing gaseous intermediates detected by the MS system. A prime contender for this would be adsorbed species on the catalyst surface, but there also might be some degree of soluble species which are not detected. The CO evolves almost at the same time as H_2_, as a typical product of the CO_2_RR on Au. The Tafel slopes of the faradaic current and CO MS response follow each other over about one order of magnitude with a value of 395 mV decade^−1^. With the decrease of potential below −0.6 V, the production rates of CO and H_2_ start to deviate, as the former is slowly increased while the latter is dramatically improved, with a slope of 153 mV decade^−1^ observed over 3 orders of magnitude. The changes seen in estimated Tafel slopes might reflect the variations in rate‐determining steps or proton donor environment for the electrocatalytic processes on Au.[^31^, ^32^, ^33^] In the lower potential region below −0.9 V, the MS response of CO gradually reaches a plateau, indicating the generation of CO becomes less favoured. Meanwhile, the MS response of H_2_ tends to follow the faradaic current again. These results are in good accordance with the literature.^[34]^ Moreover, as H_2_ and CO are the two main products for the CO_2_RR on Au, the faradaic efficiencies (FEs) can be estimated by comparing the sEC‐MS data recorded in N_2_ and CO_2_ (see Section S4 in the Supporting Information for more details and Figures S13–S14).^[20]^ As shown in Figure S15, the FEs derived from the sEC‐MS‐SI measurements agree well with those using GC presented in this work and reported elsewhere.[^35^, ^36^] Note that these behaviours are obtained from the GAME, which is in a static state yet equivalent to the case of rotating disk electrode (RDE) setup where high‐rate rotation is usually used. Thus, the results in turn highlight the fast mass transport facilitated by the GAME design and also confirm the excellent sensitivity of the sEC‐MS‐SI configuration to elucidate the dynamic product evolution under potential/current‐varying conditions.

A further benefit of our electrode system compared to other work in the literature is that the reactant CO_2_ is kept in gas phase and not required to be dissolved in the electrolyte apart for the last 100 nm or so,^[37]^ leading to much higher transport rates of CO_2_ to the catalyst surface and concomitant reduced transport time. This approach is much more similar to what would occur within an operational electrolyser. This is unlike many other approaches which rely on dissolving CO_2_ within the electrolyte and flowing that electrolyte over the electrode surface.[^9^, ^17^, ^18^, ^20^, ^21^] In our approach, not only is CO_2_ transport from the gas phase to the electrocatalyst fast, but also the transport from the electrocatalyst to the gas phase is rapid, as the product species only needto transit ca. 100 nm of electrolyte before reaching a gas pore. This not only means that species transport is facilitated, but also indicates that possible bubble formation is mitigated. Indeed, we have shown that similar electrodes to those used in this study can operate in hydrogen evolution at 6 A cm^−2^ geometric current density without bubble formation.^[38]^ In contrast, in conventional approaches the MS probe must be positioned above the electrode surface with a desire to keep this value as small as possible to improve the collection efficiency of the detection system but typically limited to a minimum of 100–200 μm of electrolyte (i.e., 1000 times farther than the distance molecules have to diffuse through in our electrode structure to reach a gas pore). The transit time across 100–200 μm distance of electrolyte will still require 10’s of seconds assuming typical diffusion coefficients of low molecular weight molecules in aqueous electrolytes ($t_{diff}∼{\left(\ell /2\right)}^{2}/D$ ), but more crucially the response will be “blurred” by the diffusion process, leading to loss of time resolution in the response (this blurring will be featured by an equivalent period to the diffusion time). Variation in the separation between the MS probe and the electrode surface will then affect this time delay (and the degree of blurring) in a highly nonlinear manner. Moreover, there is a final complexity in utilising the MS probe configuration which is poorly discussed in the literature ‐ the effect that the MS probe has on the local mass transport, current distribution and potential profile of the electrode surface. This effect potentially generates large errors. It is well established that positioning probes (e.g., Luggin capillaries) very close to the working electrode can lead to perturbations due to the “shielding” effect the probe has on the working electrode immediately coincident with that probe. In order to reduce this effect and not perturb the potential experienced by the electrode opposite to the probe, the separation should be at least twice the probe diameter,^[39]^ but this value is usually never met. However, such an effect does not occur with our GAME electrode as the side on which the reactant is collected is on the other side of the electrolyte, so there is no possibility of shielding.

**Figure 6:**
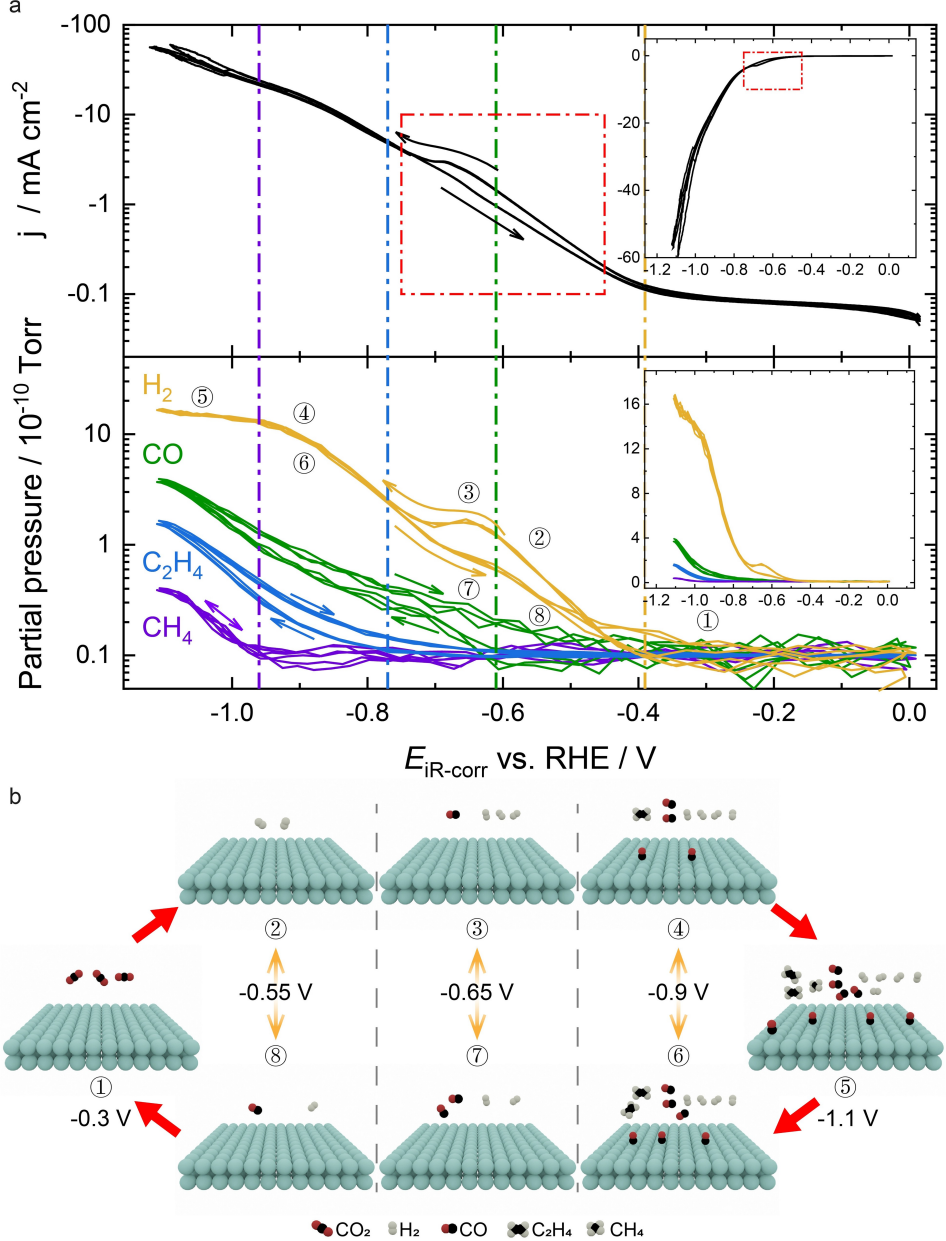
(a) CO_2_RR performance of Cu−Au/PCTE electrode showing the overlaid results of three consecutive cyclic voltammograms (5 mV s^−1^). The top panel shows the electrochemical current density on a logarithmic current scale. The lower panel shows the partial pressures of H_2_ (*m/z*=2) and CO (*m/z*=28); C_2_H_4_ (*m/z*=26) and CH_4_ (*m/z*=15) determined by sEC‐MS‐SI, under an environment of CO_2_ in 0.5 M KHCO_3_. These data are also plotted on a linear current scale, shown in the insets. The vertical lines show the onsets of gas evolution during the scan in the negative‐going direction. (b) Schematic illustration of the dynamic states of the surface and the coverage variations of product species at different potentials in the cathodic and anodic sweeps of cyclic voltammetry.

Copper has been regarded as a unique metal that favours the production of hydrocarbons, due to the moderate binding strength with many intermediates.[^16^, ^18^, ^26^, ^40^, ^41^] In this regard, we looked at the performance of Cu by electrodepositing it ontoAu/PCTE to obtain the bimetallic electrode of Cu−Au/PCTE, providing an ideal catalyst to examine the capability of sEC‐MS‐SI for elucidating various CO_2_RR products. Figure 6a shows the CO_2_RR performance of Cu−Au/PCTE electrode (also see Figure S16), demonstrating the simultaneous measurements of faradaic current, H_2_, CO, C_2_H_4_ and CH_4_ traces as a function of applied potential. The top and bottom panels show the faradaic current and the MS data with a logarithmic y‐axis, respectively (linear y‐axis scale in the insets). Three consecutive scans are displayed and the results overlay one another with a high degree of repeatability, thereby showing no evidence of artefacts due to slow diffusion of reactants to the MS. There are a number of interesting features seen in the plots, which are better visualised using a logarithmic scale to the sEC‐MS‐SI data. The faradaic current hardly shows any hysteresis, except in the potential range of −0.45 to −0.75 V (red dashed box). The MS signals of H_2_, CO and C_2_H_4_ show some hysteresis, while the CH_4_ response does not. The hysteresis in the MS response is not due to an instrumental artefact (note that no hysteresis is seen for the H_2_ signal at low potentials nor the CH_4_ signal), but rather associated with changes in the surface activity towards the electrocatalytic reaction in response to the surface coverage of intermediates and products. For H_2_, CO and C_2_H_4_, the onset potentials (highlighted by the vertical lines; −0.39 V for H_2_, −0.61 V for CO, −0.77 V for C_2_H_4_), at which gas evolutions are detected during the negative‐going scan are more negative than those in the reverse scan, respectively. For CH_4_, there does not seem to be any difference between the forward and back scans with the gas evolution starting at −0.97 V. These results agree well with previous studies.[^18^, ^42^]

Notable is the hysteresis loop for the hydrogen evolution observed in the potential range of −0.45 to −0.75 V (bottom panel of Figure 6a), aligned well with that for faradaic current. As seen in the MS responses, this loop is associated with an initial rapid increase in hydrogen evolution, followed by a peak (formed at the potential where CO evolution commences) as the potential is decreased, and then a less rapid rise in hydrogen evolution as the potential further decreases—i.e., at lower potentials the surface becomes less activated towards the HER. In the anodic scan, the process is reversed with a relative acceleration in the HER occurring in the region where the CO evolution reaction is ceasing. The HER also shows a plateau at low potentials—this limitation may be associated with “crowding” of the surface by reaction intermediates of the CO_2_RR, hindering the further acceleration of HER.

During the anodic scan, the MS responses of both CO and C_2_H_4_ are higher than their counterparts in the cathodic scan, and the gas evolutions continue to higher potentials, asymptotically approaching the baseline about 200 mV higher than the onset potentials mentioned above. This is also partially seen for the H_2_ trace as discussed above but not for the CH_4_ trace. However, as the following scan in the negative direction retraces the previous scan, the hysteresis is not derived from a permanent modification of the surface but rather due to a dynamic effect on a time scale of several to 10’s seconds. One possibility for this effect would be associated with a local pH increase at the electrode surface, as a result of the consumption of protons (or the generation of OH^−^ions) during CO_2_RR.[^25^, ^43^, ^44^] This local change further leads to a shift in the equilibrium potential of the reaction. At high potentials where no reactions occur, the pH would then relax back to bulk values. However, if this were the case, it might be expected that HER would show a severe distortion and a large hysteresis at high current densities, which is not seen. Hence, a possible local pH shift is unlikely to be the intrinsic reason. An alternative hypothesis is that the surface coverage of intermediate reactive species requires some time to equilibrate with the applied potential, i.e., the system is not under dynamic equilibrium at the scan rate used. This behaviour is in good accordance with previous studies, where the hysteresis in C_2_H_4_ generation is ascribed to the accumulation of CO (increasing linearly with negative potentials on Cu^[45]^) during the cathodic scan, which can also hinder the H_2_ production.[^16^, ^18^, ^30^]

The hypothesis is further illustrated with a cartoon in Figure 6b, showing how the surface coverage of some key species might change with representative potentials during the CVs. At each potential, there are a pair of states (−0.55 V: ②&⑧; −0.65 V: ③&⑦; −0.9 V: ④&⑥), which correspond to unsteady‐state surfaces, with the dynamically equilibrated steady‐state surface being intermediate between these two states. This would suggest that if the scan rate is decreased then the hysteresis loops should close, and the steady state surfaces could be obtained at each of these potentials. Some clues can be derived from a previous work, where the width of the hysteresis loop is reduced as the scan rate is lowered from 10 to 1 mV s^−1^.^[30]^ At less negative potentials, the reaction process is dominated by HER, generating H_2_ as the main product. As the potential is decreased, the CO* coverage is increased, while the binding strength of Cu‐CO* is weakened. This can have twofold effects: leading to the CO evolution and lowering the CO* dimerisation barrier to form C_2_H_4_.[^42^, ^46^] It is also noteworthy that the CH_4_ evolves from the potential at which the H_2_ response plateaus. This is possibly because that at larger overpotentials, the higher H coverage facilitates the hydrogenation of a C1 intermediate to CH_4_.^[47]^ This interesting hypothesis and the dynamic transition to steady state will be explicitly examined in our future work.

## Conclusion

In this work, the activity and selectivity of CO_2_RR on Au and Cu have been extensively examined by the correlative sEC‐MS‐SI technique. We are able to effectively correct for instrumental delay times and eliminate the instrumental broadening effects due to slow diffusion associated with conventional MS techniques. By introducing a selective soft ionisation strategy, we are able to accurately assess CO formation **without** the interference of reduced ionisation energy on other fragments, meaning that we can optimise the detection of all relevant species allowing for the detection of CO in the presence of CO_2_ along with other products. Therefore, the efforts devoted herein lead to a high‐resolution assessment of the dynamic evolution of CO_2_RR products, generating a real picture of the reaction mechanism and kinetics. The synchronous results reveal the onset potentials for the products in an order of H_2_>CO>C_2_H_4_>CH_4_, and the hysteresis observed for certain species in the scans can be attributed to the coverage variance of reaction intermediates (e.g., CO*). This work not only sheds light on the instrumental coupling for synchronised characterisation, but also provides significant insights towards the understanding of complex reaction pathways.

## Conflict of interest

The authors declare no conflict of interest.

1

## Supporting information

As a service to our authors and readers, this journal provides supporting information supplied by the authors. Such materials are peer reviewed and may be re‐organized for online delivery, but are not copy‐edited or typeset. Technical support issues arising from supporting information (other than missing files) should be addressed to the authors.

Supporting Information

## Data Availability

The data used in the preparation of the figures in this paper are available at DOI:(DOI inserted at proofing stage)
