# Supplementary material for: Time‐Resolved Product Observation for CO2 Electroreduction Using Synchronised Electrochemistry‐Mass Spectrometry with Soft Ionisation (sEC‐MS‐SI)
Source: Angew Chem Int Ed Engl. 2023 Oct 24;62(48):e202312607. doi: 10.1002/anie.202312607 (PMC10952920; doi:10.1002/anie.202312607)
Supplement: Supplementary file 1 — Supporting Information [file ANIE-62-0-s001.pdf]

Angewandte Chemie
Eine Zeitschrift der Gesellschaft Deutscher Chemiker

Supporting Information

Time-Resolved Product Observation for CO_2 Electroreduction Using Synchronised Electrochemistry-Mass Spectrometry with Soft Ionisation (sEC-MS-SI)

G. Zhang, A. Kucernak**

Supporting Information
©Wiley-VCH 2021
69451 Weinheim, Germany

Time-Resolved Product Observation for CO_2 Electroreduction Using Synchronised Electrochemistry-Mass Spectrometry with Soft Ionisation (sEC-MS-SI)

Guohui Zhang,*[a,b] Anthony Kucernak*[a]

[a] Department of Chemistry, Imperial College London, White City Campus, London W12 0BZ, United Kingdom

[b] State Key Laboratory of Catalysis, Dalian Institute of Chemical Physics, Chinese Academy of Sciences, Dalian 116023, China

*Correspondence: gzhang@dicp.ac.cn; anthony@imperial.ac.uk

Table of Contents

S1| Experimental 2
S2| Fragmentation and ionisation of gaseous species in the mass spectrometer........ 4
S3| Cyclic and chronoamperometric curves of Au/PCTE and corresponding MS responses 6
S4| Correlation of electrochemical and MS responses for Au/PCTE and Cu-Au/PCTE 9
References........ 12

S1| Experimental

Electrochemical measurements were performed with an Autolab potentiostat (PGSTAT302N, Metrohm). A thin layer of gold (~100 nm) was deposited onto a polycarbonate track etch (PCTE; Sterlitech, PCTF0447100) membrane using a Quorum sputter coater (Q300 TD). The as-prepared sample, denoted as Au/PCTE, was thoroughly cleaned in isopropanol and DI water Soxhlet, respectively, each for 8 h, and then left to dry in clean air. The membrane was then cut into the appropriate sizes and mounted onto the GAME. For the production of the bimetallic electrode of Cu-Au/PCTE, the GAME was also kept at -2 V for 20 s (against a Cu wire) in a solution containing 0.2 M $CuSO_4$ and 1.5 M H_2SO_4, as descried in our previous work.[1-2] In the CO_2RR measurements, a solution of 0.5 M $KHCO_3$ was used as the electrolyte in a gas-tight glass cell with a PTFE lid. Prior to the experiment, the solution was sparged with N_2 for >30 min to remove the residual O_2. The XYZ movements of the GAME in the electrochemical cell were manipulated by a custom-built holder. When in contact with the solution, the Au/PCTE or Cu-Au/PCTE substrate on the GAME acted as the working electrode (WE), with an exposed electrode area of ca. 0.33 cm^2. A leak-free Ag/AgCl and a gold coil served as the reference electrode (RE) and the counter electrode (CE), respectively, which were situated in separate compartments, with the RE placed close to the WE via a Luggin capillary.

The mass spectrometric responses were recorded on a Hiden QGA (Hiden Analytical, Warrington, England) system, with data acquisition controlled with the software of MASsoft 7. An electron energy of 70 eV and an emission current of 200 μA were used for detecting the species, while an energy of 17 eV was locally created for evaluating the signals at *m/z*=28 (vide infra). An event sequence was used in MASsoft 7 to comply with the commands sent from the potentiostat, leading to a synchronisation format.

The instrumental setup of the sEC-MS-SI is illustrated in Figure S1a. The communications between the potentiostat and the MS consist of two sequences: one for sending the trigger command (‘ON’ to start or ‘OFF’ to stop), the other for transferring the electrochemical signals (e.g., current and potential) to the MS interface. Therefore, this arrangement allows the MS to be simultaneously operated and controlled by the potentiostat, while the electrochemistry can be aligned with the MS at the same sampling rate. The working principle of the sEC-MS-SI is further shown in Figure S1b. Prior to the start of the electrochemical measurements, the MS will be set into a ‘Standby’ status. Upon receiving a trigger ‘ON’ signal from the potentiostat, the MS starts to record signals of the products generated from the electrochemical reaction. Meanwhile, the electrochemistry data is immediately imported to the MS software, and plotted with the MS responses in the same time axis. This arrangement ensures the datasets from both instruments to be simultaneously initiated and recorded at the same sampling rate, thereby providing a direct view of the possible delay time associated with the reaction system (vide infra). When one set of electrochemical scans are finished, a trigger signal of ‘OFF’ from the potentiostat will be sent to terminate the MS and shift it back to ‘Standby’ status. The subsequent action of the MS is fully subject to the activity of starting or stopping new electrochemical measurements on the potentiostat. The cables and connectors for configuring the sEC-MS-SI are shown in Figure S1c-d, and the detailed pin assignments are illustrated in Table S1.

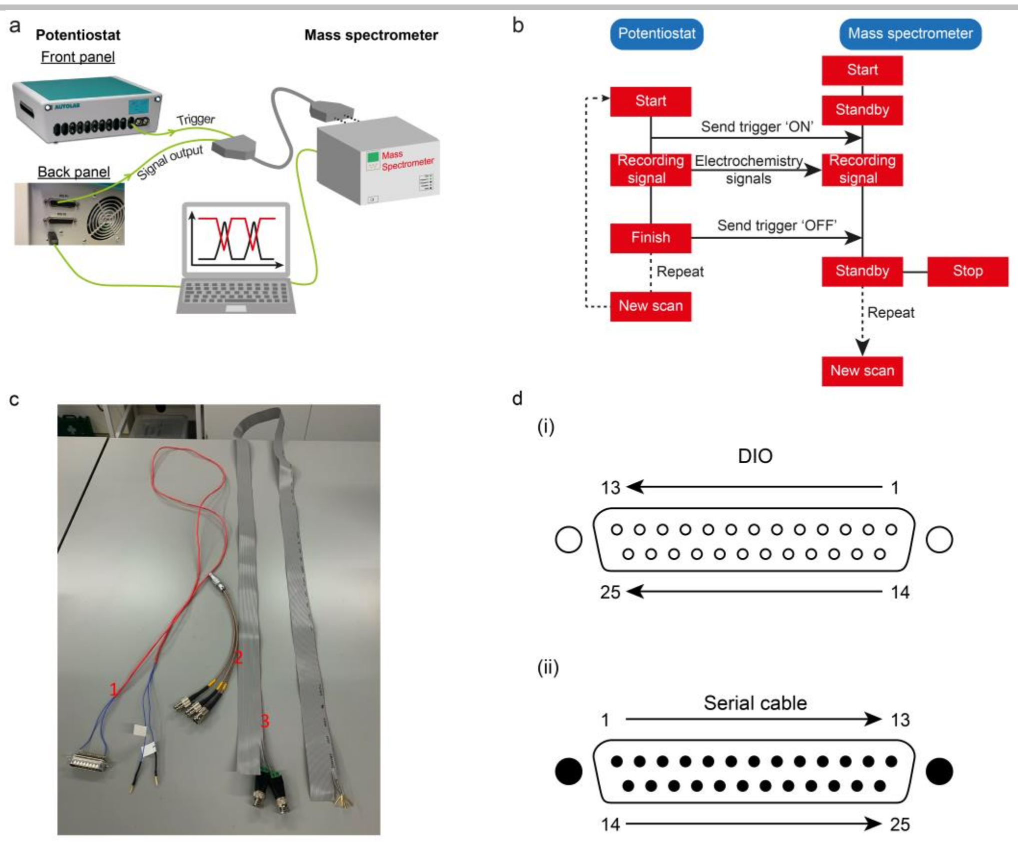

Figure S1. (a) Schematic for the hardware connections of the sEC-MS-SI platform. (b) Outline of the workflow for the synchronisation of sEC-MS-SI. (c) Custom-made connection cables for different functions: (1) a trigger cable including a 25 -pin D-sub connector, (2) a commercial monitor cable connector and (3) a flat ribbon cable connected with 2 BNC adapters for sending electrochemical signals (e.g., voltage, current) to the MS. (d) Pin arrangements for (i) the female connector of Autolab potentiostat and (ii) the male serial cable connected to the Hiden QGA spectrometer.

Table S1. Connection details for the synchronization of electrochemistry and mass spectrometry.

**Function from potentiostat**	**Pin No. / BNC adapter part**		
	Potentiostat		Mass spectrometer
Trigger	Back panel (via D-sub connector)	1	10
		25 (ground)	12
Signal output (voltage)	Front panel (via the trigger cable)	Connector body	4
		Center pin	17

The CO_2RR products on Au/PCTE were also quantified by coupling the GAME with a GC (SRI Multiple Gas Analyser MG#5). The GC was equipped with a flame ionisation detector (FID) along with a methaniser for CO detection and a thermal conductivity detector (TCD) for H_2 evaluation. Argon gas (BOC UK, N 6.0) was used as the mobile phase and a gas mixture of 97% Ar, 2% H_2 and 1% CO was adopted for calibration. The FE of a certain species, *i*, can be obtained from the equation below:

$$FE_i = \frac{x_i \times n \times F \times v}{V \times j_{tot}} \times 100\%$$

where x_i is the volume faction of product *i*, *n* is the number of electrons needed to produce 1 mole *i*, *F* is Faraday's constant and *v* is the flow rate, *V* is the molar volume of gas, and j_{tot} is the total current density.

S2| Fragmentation and ionisation of gaseous species in the mass spectrometer

As seen in Figure S2, when ionised by MS, CO_2 produces a signature peak at *m/z*=44, and the rest of the peaks are due to the fragmentation. Of note, the presence of these secondary peaks can adversely interfere with the detection of species targeted at the same *m/z* values. As an example, the cracking pattern of CO molecule is shown in Figure S3. The primary peak is located at *m/z*=28.

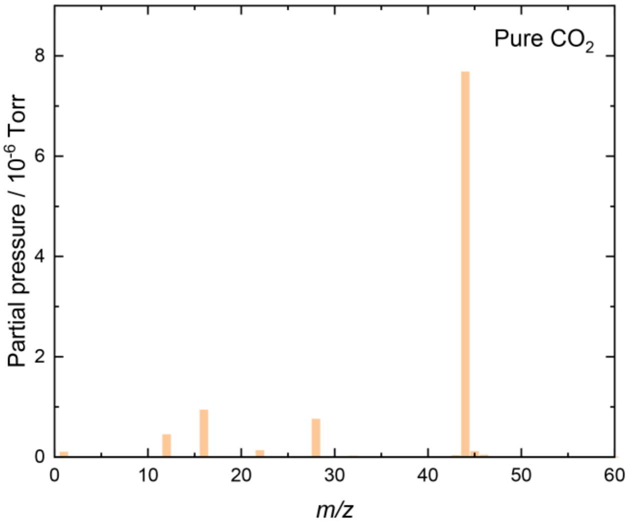

Figure S2. Mass distribution profile of cracked CO_2 using default MS configuration.

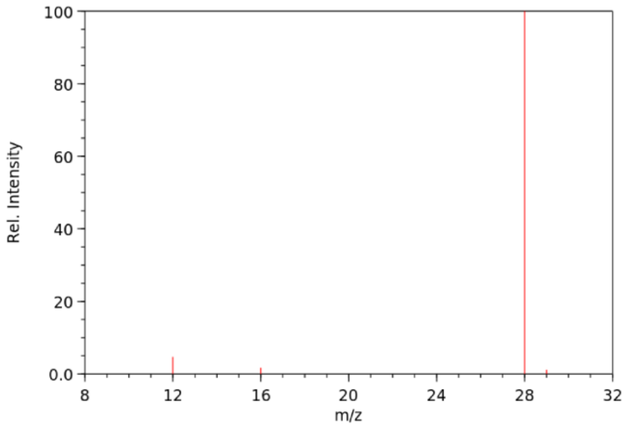

Figure S3. Mass distribution profile of cracked CO using default MS configuration.[3]

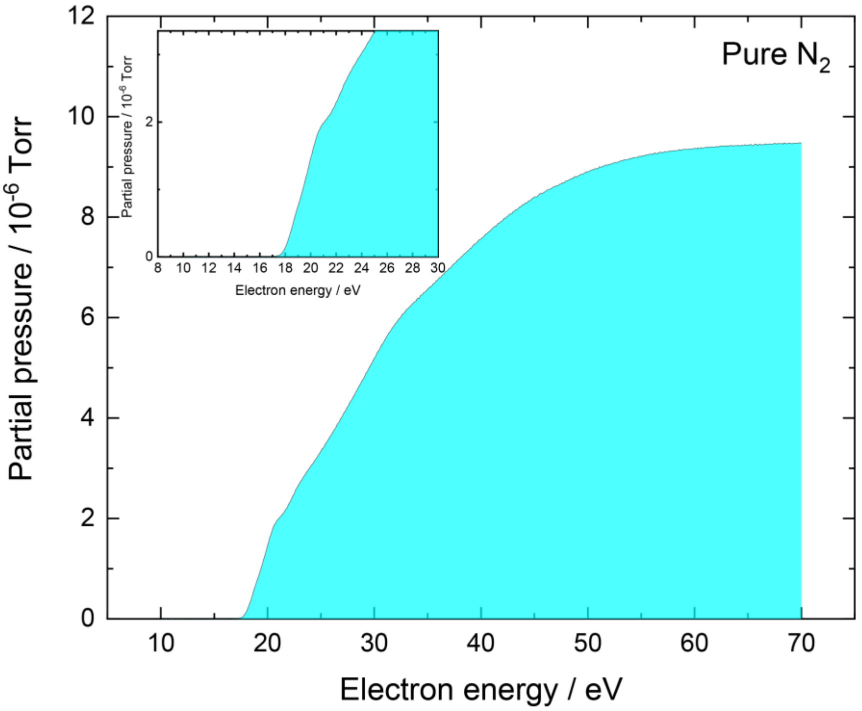

Figure S4. MS responses of *m/z*=28 (in this case N_2^+) as a function of electron energy of the mass spectrometer under N_2 atmosphere.

S3| Cyclic and chronoamperometric curves of Au/PCTE and corresponding MS responses

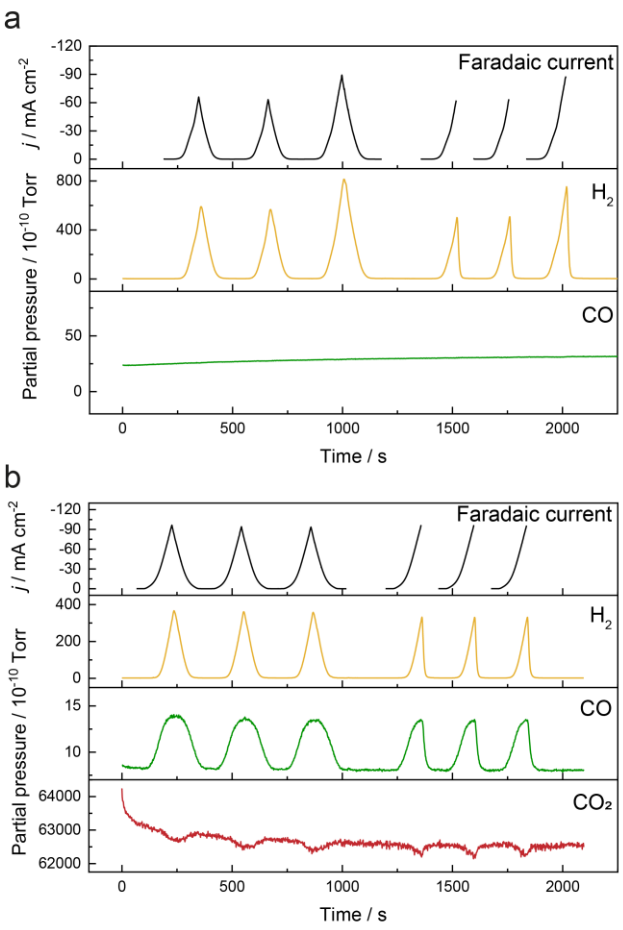

Figure S5. Faradaic current and mass spectrometric responses for the products of Au/PCTE cycled in (a) N_2 and (b) CO_2, respectively. Scan rate: 5 mV s^{-1}.

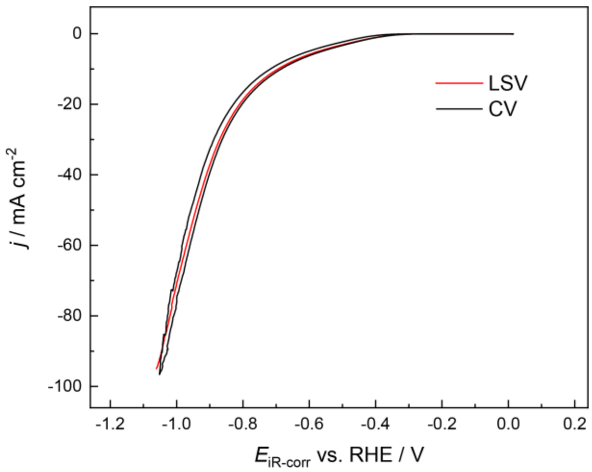

Figure S6. Comparison of the LSV and CV of Au/PCTE recorded under CO_2 in 0.5 M $KHCO_3$. Scan rate: 10 mV s^{-1}.

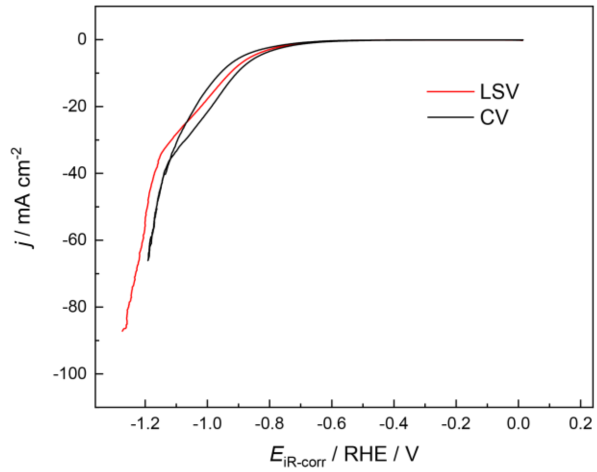

Figure S7. Comparison of the LSV and CV of Au/PCTE recorded under N_2 in 0.5 M $KHCO_3$. Scan rate: 10 mV s^{-1}.

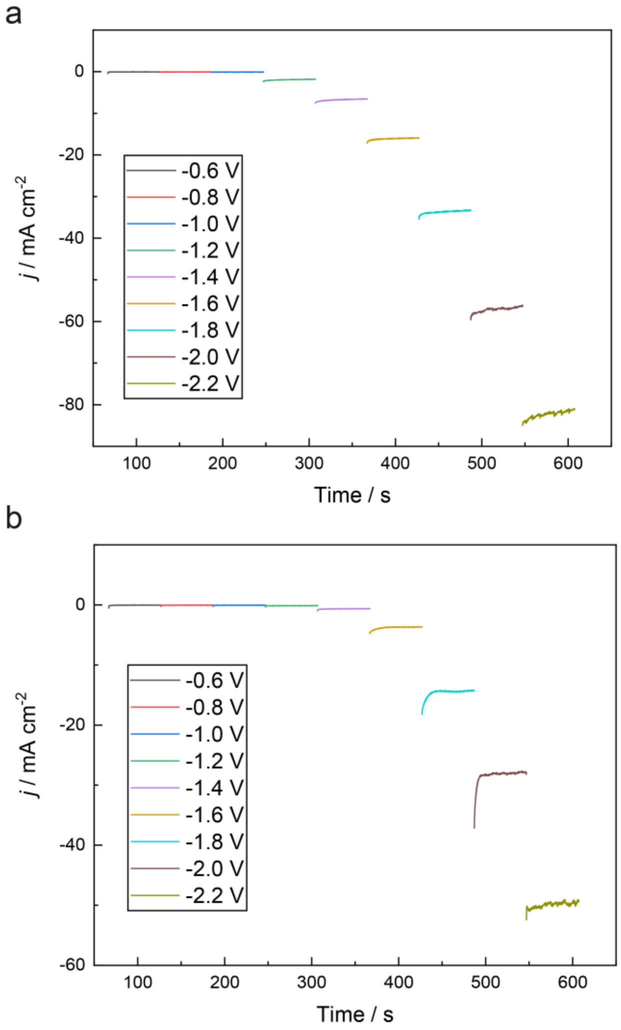

Figure S8. Chronoamperometry of Au/PCTE recorded in (a) CO_2 and (b) N_2, respectively. Note that the potentials in the legends are not iR corrected.

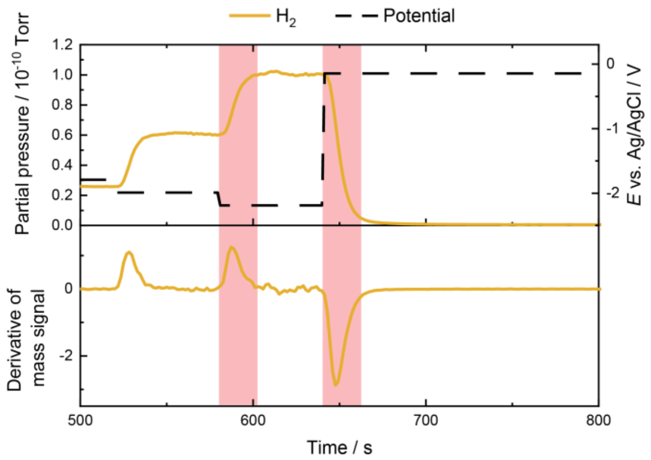

Figure S9. Potential profile and MS response of H_2 during the chronoamperometry of Au/PCTE in 0.5 M $KHCO_3$.

S4| Correlation of electrochemical and MS responses for Au/PCTE and Cu-Au/PCTE

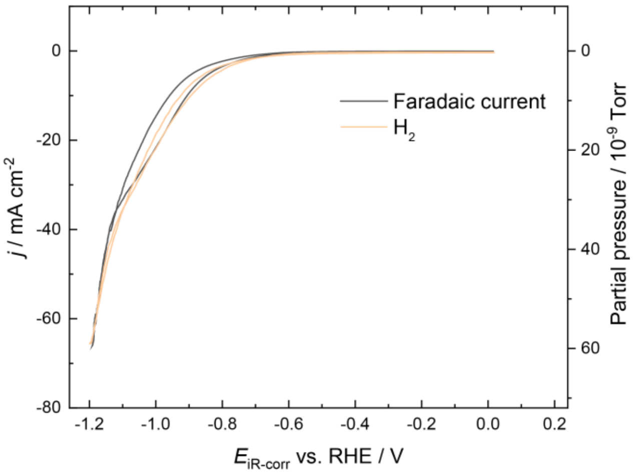

Figure S10. CV (recorded at 10 mV s^{-1}) and the corresponding MS response of H_2 (*m/z*=2) for Au/PCTE in 0.5 M $KHCO_3$ under N_2 atmosphere.

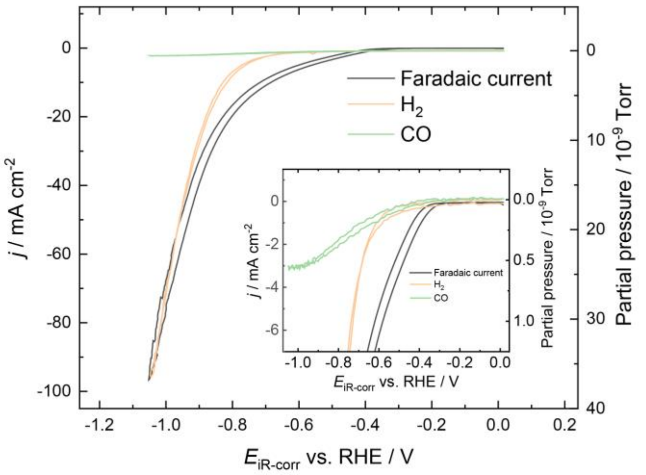

Figure S11. CV (recorded at 10 mV s^{-1}) and the corresponding MS responses of H_2 (*m/z*=2) and CO (*m/z*=28) for the CO_2RR of Au/PCTE in 0.5 M $KHCO_3$. The zoom-in area is shown in inset.

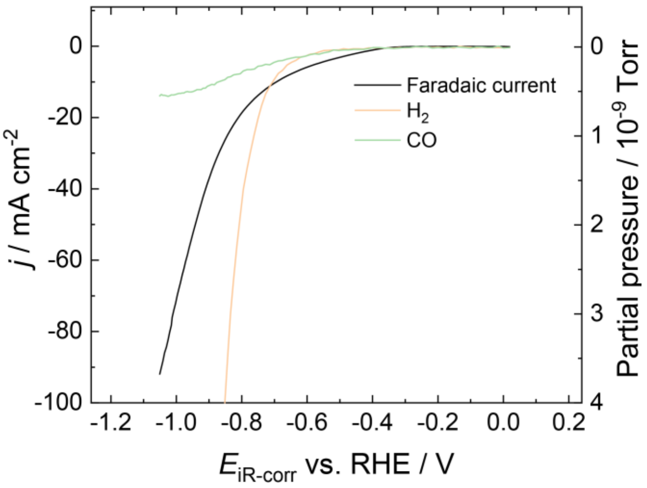

Figure S12. Enlarged view of the inset in Figure 5c in the main text.

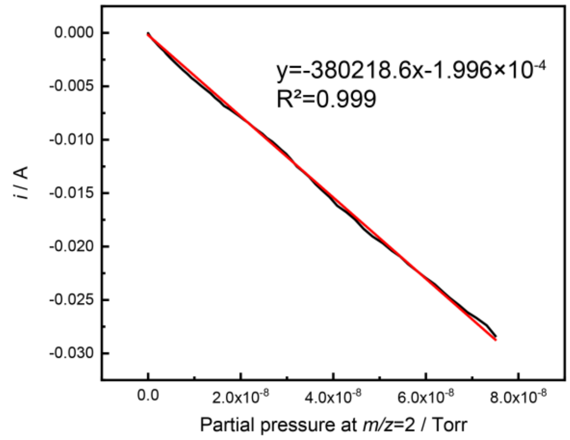

Figure S13. Plot of faradaic current as a function of H_2 MS response (*m/z*=2), during the LSV (10 mV s^{-1}) recorded in 0.5 M $KHCO_3$, under N_2 atmosphere (shown as the inset in Figure 5b of the main text).

The FEs for the CO_2RR products were estimated from the sEC-MS-SI data recorded in the absence and presence of CO_2, as done in a previous work.[4] In short, a linear relationship between faradaic current and MS response (*m/z*=2) was derived from Figure 5b in the main text (Figure S13), since the LSV recorded in N_2 is assumed to be purely due to the HER. Then, this H_2 calibration curve was used to calculate the H_2 partial current from the overall faradaic current observed in CO_2. As H_2 and CO were the two main products for the CO_2RR on Au, the CO partial current was also obtained. The FEs for H_2 and CO are shown in Figure S14, which are in good agreement with the GC measurements using the GAME, as well as those reported by Hori *et al.* (Figure S15).[5]

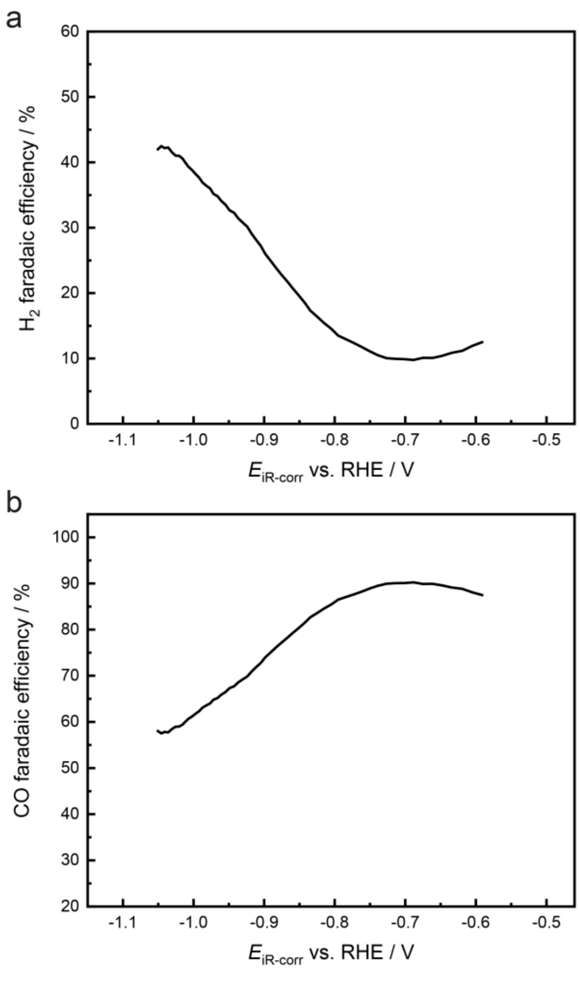

Figure S14. (a) H_2 and (b) CO faradaic efficiency derived from the sEC-MS-SI measurements of Au/PCTE during LSV (10 mV s^{-1}) conducted in 0.5 M $KHCO_3$, under CO_2 atmosphere.

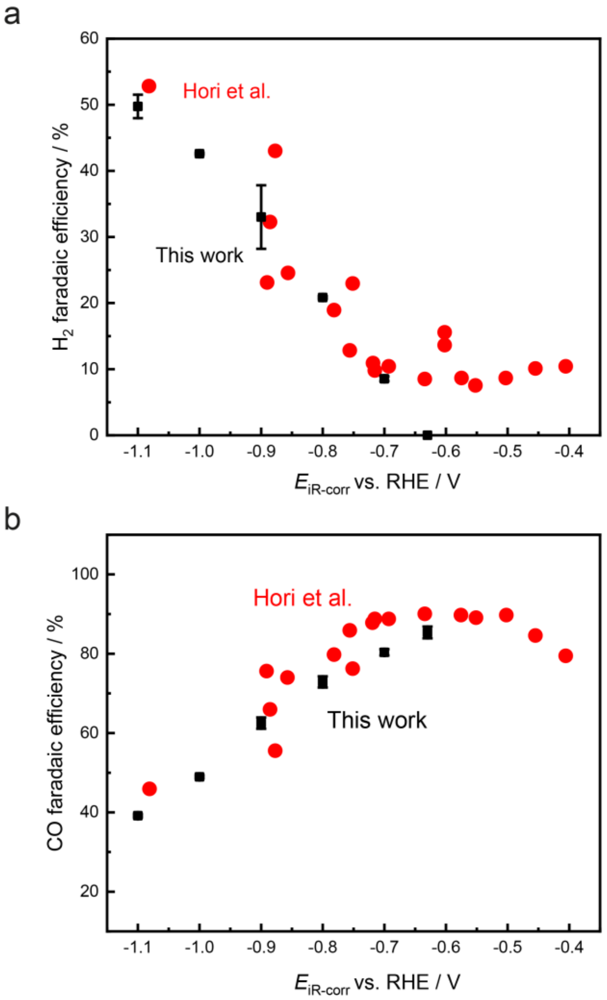

Figure S15. (a) H_2 and (b) CO faradaic efficiency derived from the GC measurements during chronoamperometry steps applied to Au/PCTE in 0.5 M $KHCO_3$, under CO_2 atmosphere. Error bars (for 2 measurements) are indicated. As a comparison, the data reported by Hori *et al.*[5] from the CO_2RR on Au in 0.5 M $KHCO_3$ is shown in red.

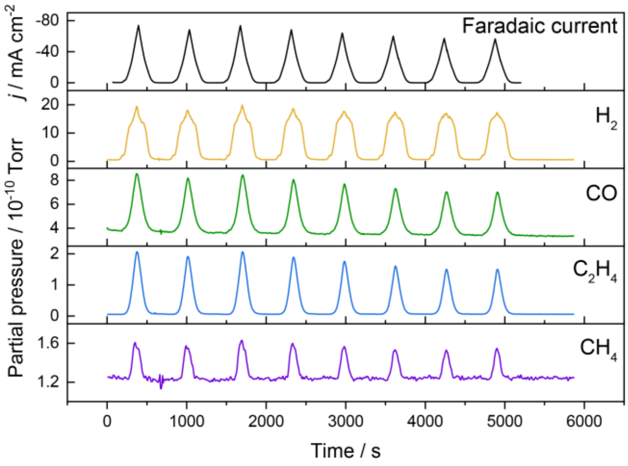

Figure S16. Faradaic current and mass spectrometric responses for the CO_2RR products of Cu-Au/PCTE. Scan rate: 5 mV s^{-1}.

References

[1] G. H. Zhang, Y. X. Cui, A. Kucernak, *ACS Catal.* **2022**, *12*, 6180-6190.
[2] G. H. Zhang, A. Kucernak, *ACS Catal.* **2020**, *10*, 9684-9693.
[3] *NIST Chemistry WebBook*, https://webbook.nist.gov/chemistry.
[4] K. Ye, G. Zhang, X.-Y. Ma, C. Deng, X. Huang, C. Yuan, G. Meng, W.-B. Cai, K. Jiang, *Energy Environ. Sci.* **2022**, *15*, 749-759.
[5] H. Yoshio, M. Akira, K. Katsuhei, S. Shin, *J. Chem. Soc., Chem. Commun.* **1987**, 728-729.
